# Changes in Seasonal Spatial Distribution Patterns of *Euprymna berryi* and *Euprymna morsei*: The Current and Predictions Under Climate Change Scenarios

**DOI:** 10.3390/biology14040327

**Published:** 2025-03-24

**Authors:** Min Xu, Yong Liu, Xiaojing Song, Linlin Yang

**Affiliations:** 1 Key Laboratory of East China Sea Fishery Resources Exploitation, Ministry of Agriculture and Rural Affairs, Shanghai 200090, China; xuminwzy@aliyun.com (M.X.); liuy@ecsf.ac.cn (Y.L.); songxiaojing@ecsf.ac.cn (X.S.); 2 East China Sea Fisheries Research Institute, Chinese Academy of Fishery Sciences, Shanghai 200090, China

**Keywords:** ecological succession, Sepiolid, paralarvae, T-S diagram, nekton community, nursery, spawn, Yellow Sea, bobtail squid

## Abstract

Given their small size and low value in commercial fishing and aquaculture, little is known about the seasonal spatial distribution patterns and characteristics of the bobtail squid *Euprymna berryi* and *Euprymna morsei* in seas around China. Thus, we conducted seasonal bottom-trawling surveys in the southern Yellow and East China Seas during 2018 and 2019. Our results showed that *E. berryi* migrated from inshore areas (Yushan fishing grounds) during the summer and autumn to offshore areas (Wentai fishing grounds) during the winter. In contrast, *E. morsei* migrated from shallower water areas during the spring to deeper water areas during the winter. The highest abundance of *E. berryi* versus *E. morsei* was found in areas where temperatures were 25.29–28.02 °C compared with 19.54 °C (33.43–34.04‰ versus 34.43‰), respectively, during the summer; 20.99–21.69 °C compared with 21.98–22.70 °C (34.07–34.50‰ versus 33.80–33.60‰), respectively, during autumn; and 17.13–20.36 °C compared with 10.51–13.49 °C (34.23–34.46‰ versus 31.69–33.42‰), respectively, during winter. We predict that suitable habitats for *E. berryi* would expand more northward under SSP585-2050, whereas those for *E. morsei* would shrink into more northern locations under SSP370-2100 and SSP585-2100. The SSP245-2100 and SSP585-2100 scenarios had the most negative impacts on the distributions of both species. Such insights improve our understanding of the population dynamics and habitat requirements of both species to support their population management and exploitation in response to future climate change.

## Simple Summary

Climate change has the potential to trigger the ecological succession of species and communities. In this study, we used ten algorithms (artificial neural network, classification tree analysis, flexible discriminant analysis, generalized additive model, generalized boosting model, generalized linear model, multiple adaptive regression splines, random forest, surface range envelope, and extreme gradient boosting training) to construct ensemble models to predict the variation in habitat distribution of the bobtail squid *Euprymna berryi* and *Euprymna morsei* under different climate scenarios (SSP1-2.6, SSP2-4.5, SSP3-7.0, and SSP5-8.5) and across different seasons in the East China Sea region. Our results indicated that *E. morsei* would experience larger negative impacts under the different climate scenarios compared with *E. berryi*, highlighting the potential for species succession. Such insights require further research on the biological and ecological aspects of the small-sized squid *E. berryi* and *E. morsei*. Fisheries managers also need to consider the incorporation of climate change scenarios in current fisheries’ policy-making and management plans.

## 1. Introduction

Scientists have long-term observed the relationship between species distribution and the environment, and climate-induced changes might cause variations at the levels of individual species and entire communities [1,2]. Climate, especially temperature, is a primary driver of biological processes [3]. Climate warming is impacting many aspects of animal and plant communities and, thus, regional ecosystems, such as shifts in the geographical distribution range and succession of species [4]. Such changes could increase the risk of species extinction, alter community structure, and disrupt ecological interactions and ecosystem functions [5,6,7]. Successional changes in plant communities in foredune, interdune, and backdune habitats were reported in response to climate disturbances [8], while experimental warming led to divergent succession of soil microbial communities [9]. Climate change could also cause changes in the proportional variation in pelagic species in marine environments [10]. Thus, there is a need to observe the habitat distribution variations in area range of both species from the same genus in the same regional area to identify potential species succession under different global warming scenarios.

The East China Sea region, including the southern Yellow and East China Seas, is located at the margin of the Northwest Pacific Ocean [11]. This area is subject to significant atmospheric and oceanic variability on interannual to decadal timescales driven by large-scale coupled ocean–atmosphere variability, such as the Arctic and El Nino-Southern Oscillations [12]. From 1958 to 2018, this area experienced continuous rapid warming, with the sea surface temperature increasing by ~0.98 ± 0.19 °C, higher than the global ocean average temperature change (0.54 ± 0.04 °C) [12]. In the Mediterranean Sea, the sea surface temperature has increased by 1.5 °C from 1982 to 2018 [13,14]. Under different climate scenarios, such as Shared Socioeconomic Pathway (SSP) 1–2.6 and SSP5-8.5, this ocean warming will continue, accompanied by sea-level rises and more frequent extreme sea-level events. Such abiotic changes will also trigger significant changes in the biota of this region. Thus, it is vital to identify how different climate scenarios could change the distributions of aquatic species.

Sepiolid squid, including bobtail squid of the genus *Euprymna* [e.g., hummingbird bobtail squid *Euprymna berryi* (Sasaki 1929) and morse bobtail squid *Euprymna morsei* (Verrill 1881)], are some of the smallest cephalopods, with average mantle lengths ranging from 10 to 80 m [15] (e.g., average adult mantle length of *Euprymna scolopes* is ~25 mm [16] compared with 20–40 mm in the Thai bobtail squid *Euprymna hyllebergi* [17] and 30–40 mm in *Euprymna tasmanica* [17]). The mantle length of *E. berryi* is considerably longer than that of *E. morsei* (≤50 mm versus ≤40 mm, respectively). Bobtail squid are nocturnal cephalopods with a worldwide distribution, short life span, and benthopelagic life history [18].

This study focused on *E. berryi* and *E. morsei*, both of which are widely distributed from the coast of China into Japanese waters and the Indian Ocean [19]. Their average life span is 139 and 99 days (with a maximum of 265 and 169 days), respectively [18]. They exhibit a brief nektobenthic paralarval stage and begin to demonstrate mating behavior at 90 and 70 days post-hatching, respectively [18]. Singley (1983) observed burrowing behavior of *E. scolopes* 5–6 days post-hatching [20], while Norman (2000) reported that *E. tasmanica* hatchlings quickly settled on substrates and buried into the sand [21]. In addition, *E. berryi* and *E. morsei* are intermittent terminal spawners, spawning repeatedly once reaching sexual maturity [18]. The hatchling stage includes a planktonic phase that lasts 6–8 h before the hatchling gradually adopts a benthic habit [17]. Fishery catches of *Euprymna* spp. in the East China Sea region are discarded as bycatch because of their small size and low economic value, and thus, fishing statistics for both species are unavailable. Understanding how these species might respond to changes in their aquatic environment in response to climate change is important, owing to deficient data and their role in marine food webs.

Thus, in this study, we investigated changes in the seasonal spatial distribution patterns of *E. berryi* and *E. morsei* in the East China Sea region under different climate scenarios (SSP1-2.6, SSP2-4.5, SSP3-7.0, and SSP5-8.5) for 2040–2050 (the 2050s) and 2090–2100 (the 2090s) and their responses to environmental variables (e.g., depth, water temperature, and salinity). In addition, we predicted the percentage of habitat loss, gain, and variation (Gain%–Loss%) for both species. Such insights can not only be used to improve ocean management for these species that incorporate responses to climate change but also contribute to biodiversity conservation and policy-making efforts under climate warming.

## 2. Materials and Methods

### 2.1. Sampling and Survey Procedures

**Figure 1:**
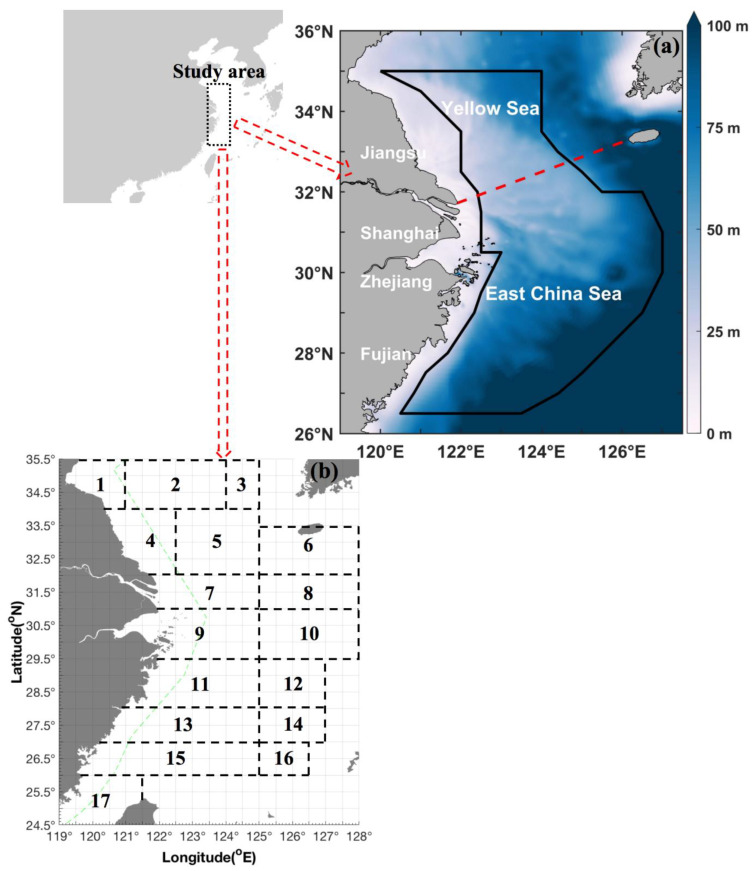
Survey map information. (**a**) Map of the study area in the East China Sea region (26.50° N–35.00° N 120.00° E–127.00° E), denoted by a dark-blue solid line. The area includes the southern Yellow and East China Seas adjacent to the coastlines of Fujian, Zhejiang, Shanghai, and Jiangsu. The color bar denotes the depth range (0 m–100 m). The red-dashed line indicates the boundary between the Yellow Sea and East China Sea. (**b**) Fishing grounds: (1) Haizhou Bay, (2) Lianqingshi, (3) Liandong, (4) Lvsi, (5) Dasha, (6) Shawai, (7) Yangtze River mouth, (8) Jiangwai, (9) Zhoushan, (10) Zhouwai, (11) Yushan, (12) Yuwai, (13) Wentai, (14) Wenwai, (15) Mindong, (16) Minwai, and (17) Minzhong.

Bottom-trawling surveys were conducted in the southern Yellow and East China Seas during 2018 and 2019. The surveys used a trawl net with a cod end mesh size of 20 mm that was towed by fisheries research vessels (Zhongkeyu 211 and 212) during the autumn (2–11 November 2018), winter (4–27 January 2019), spring (22 April–10 May 2019), and summer (13 August–27 September 2019). The study area covered 26.50°–35.00° N, 120.00°–127.00° E, and the sample trawls were conducted in a grid pattern with longitude and latitude spacings of 30 min × 30 min (Figure 1). The average trawl speed was 3 knots, and all tows were conducted for a duration of 1 h at each station. In total, 519 valid tows were included in this study (127 stations in autumn, 111 stations in winter, 141 stations in spring, and 140 stations in summer).

Environmental variables were measured at each station using a conductivity–temperature–depth profiler (SBE-19; SeaBird-Scientific, Bellevue, WA, USA). Sea surface temperature (SST), sea surface salinity (SSS), and sea surface dissolved oxygen (SSDO) were measured at 3 m below the surface, while sea bottom temperature (SBT), sea bottom salinity (SBS), and sea bottom dissolved oxygen (SBDO) were measured 2 m above the sea bottom at sea depths <50 m and at 2–4 m above the bottom at sea depths >50 m. Ottersen et al. (2010) [22] suggested that oceanographic parameters such as SST, SBT, SSS, and SBS are very important for ocean circulation patterns, vertical mixing, availability of nutrients, and subsequent marine ecosystem primary production, which appear to be the leading indicators and important drivers of marine fishery resource fluctuations.

The catches from each station were transported to the laboratory for analysis. The total number and weight of both species were counted and weighed to the nearest 0.10 g of wet weight; the catch density was calculated as the biomass density per unit of sampling time CPUE_w_ (g·h^−1^) and density per unit of sampling time CPUE_n_ (ind·h^−1^). The total values of CPUE_w_ and CPUE_n_ across the sampling sites are detailed in Table 1. The average individual weight (AIW) was defined as the CPUE_w_ divided by the CPUE_n_ at each station.

### 2.2. Ensemble Model, Selection of Environmental Variables, and Evaluations

The species distribution model is widely used as a tool for analyzing the spatio-temporal distribution patterns of marine living organisms, modelling with key steps including problem scoping, problem conceptualization, model formulation and evaluation, model application, and model perpetuation [23].

We used ten algorithms to predict the habitat distribution of both species based on the collected data: artificial neural network (ANN), classification tree analysis (CTA), flexible discriminant analysis (FDA), generalized additive model (GAM), generalized boosting model (GBM), generalized linear model (GLM), multiple adaptive regression splines (MARS), random forest (RF), surface range envelope (SRE), and extreme gradient boosting training (XGBOOST). These were combined into a species distribution model (SDM) to describe and forecast the relationship between the species and environmental variables. Araujo and New (2007) reported that combining these ten models into the SDM was more advantageous compared with single models, improving the robustness of the prediction and decreasing analysis bias, thereby yielding more confidence in the predictions [24]. We used the mean data of the surveyed four months to produce the annual model and used different seasonal data to produce the seasonal models. All the data used in the models were obtained from the surveys conducted as part of this study.

We used the ‘biomod2’ package in the ensemble SDM platform. To run the model, the data set was separated into categories of 0 (absence) and 1 (presence), and an 80%:20% split was then randomly applied for training and testing the data independently to construct the ten algorithms using the random cross-validation method [25]. The performance of each algorithm was assessed by the index of the area under the receiver operating characteristic curve (ROC) and the true skill statistic (TSS) [26]. Among these models, we selected those that performed best (a threshold value of the receiver operating characteristic curve (AUC) > 0.8) and combined them into an ensemble model using the weighted average method. The weighted average method is a method of weighting the mean of probabilities over the selected algorithms. Single algorithms are combined into an ensemble model based on the weighted average method. The single algorithm is weighted according to the obtained assessment scores.

Shared Socioeconomic Pathways (SSPs) are used to explore how societal choices will affect greenhouse gas emissions. SSPs include a world of sustainability-focused growth and equality (SSP1); a ‘middle of the road’ world where trends broadly follow their historical patterns (SSP2); a fragmented world of ‘resurgent nationalism’ (SSP3); a world of ever-increasing inequality (SSP4); and a world of rapid and unconstrained growth in economic output and energy use (SSP5) (details can be found at https://www.carbonbrief.org/explainer-how-shared-socioeconomic-pathways-explore-future-climate-change/ (accessed on 20 March 2025)).

Future climate data were obtained from CMIP6 (https://pcmdi.llnl.gov/CMIP6/ (accessed on 20 March 2025)), and predicted environmental data, such as SST, SBT, SSS, and SBS, were obtained from Bio-ORACLE (https://bio-oracle.org/index.php (accessed on 20 March 2025)). Four SSP scenarios (SSP1-2.6, SSP2-4.5, SSP3-7.0, and SSP5-8.5) for 2040–2050 (the 2050s) and 2090–2100 (the 2090s) were used in this study [27]. Bias corrections were performed for SST, SSS, SBT, and SBS, given that such correction of climate model raw data is essential to enhance the credibility of habitat distributions under future climate scenarios [28]. The delta method is a prevalent technique in fisheries habitat prediction that effectively mitigates such biases [29]. We used this approach to calculate climate differences between current and future data sets by applying corrections to the raw data. Specifically, the delta method leverages discrepancies between observed and simulated baseline conditions to adjust simulations for time (t) periods (here, 2040–2050 and 2090–2100) [30].

Bias correction for time *t* in geographical location *x* was conducted using Equation (1):

(1) $$\begin{array}{c} D_{sim}^{DM}\left(x,t\right)=D_{emp}\left(x,0\right)+\left(D_{sim}^{raw}\left(x,t\right)-D_{sim}^{raw}\left(x,0\right)\right)\\ =D_{sim}^{raw}\left(x,t\right)+\left(D_{emp}\left(x,0\right)-D_{sim}^{raw}\left(x,0\right)\right) \end{array}$$

where $D_{emp}\left(x,0\right)-D_{sim}^{raw}\left(x,0\right)$ represents the bias as the anomaly between observed and simulated environmental data at geographical location *x* and $D_{sim}^{DM}\left(x,t\right)$ denotes the bias-corrected temperature forecasts that were calculated by adding the bias to the simulated environmental data for time *t* in geographical location *x*.

## 3. Results and Discussion

### 3.1. Seasonal Variations in Environmental Variables of Both Species

Previous work recorded adult *E. berryi* occurring at a depth of up to 60 m on the Pacific side of mainland Japan [18]. In our study, *E. berryi* was found at a minimum and maximum measured depth of 55 m and 84 m, respectively, in summer and at minimum depth of 58 m and maximum depth of 107 m in autumn, indicating a potential seasonal expansion from shallower inshore to deeper offshore areas (Table 2). By contrast, *E. morsei* was found in shallower water areas in spring, expanding to the whole survey area during autumn and winter (Table 2).

The SBT ranges of *E. berryi* were similar between autumn and winter, with the highest values in autumn and winter being similar to the lowest value in summer. The SBT ranges of *E. morsei* were similar in spring, autumn, and winter, being highest from autumn to winter to spring. The SSS ranges of *E. berryi* and *E. morsei* were similar across the seasons. The SBS values for *E. berryi* were higher in summer compared with autumn and winter, a pattern also seen for *E. morsei* (Table 2). The SSDO and SBDO ranges were similar for each species in each of the seasons (Table 2).

### 3.2. Seasonal Spatial Distribution Patterns and Characteristics of CPUE_w_ and AIW

**Figure 2:**
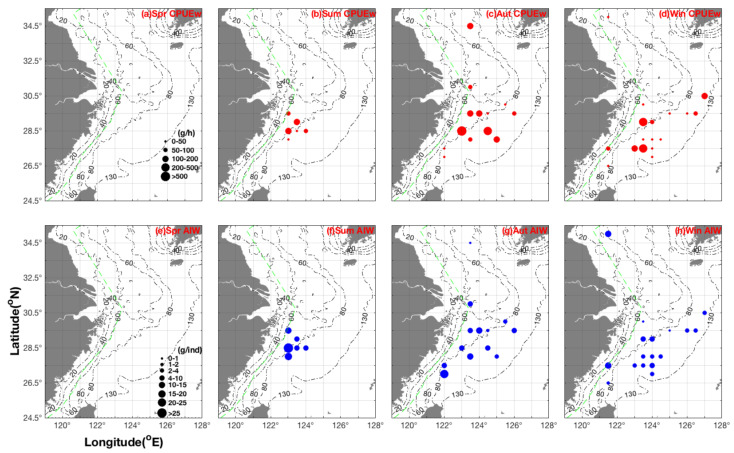
Seasonal distribution patterns of CPUE_w_ (red; grouped into 0–50, 50–100, 100–200, 200–500, and >500 g h^−1^) and AIW (blue; grouped into 0–1, 1–2, 2–4, 4–10, 10–15, 15–20, 20–25, and >25 g ind^−1^) for *Euprymna berryi*. Abbreviations: AIW, average individual weight; CPUE_w_, catch per unit effort by weight.

The highest biomass of *E. berryi* occurred in the Yushan fishing ground in summer, with a higher biomass in northern than in southern Yushan. During the summer, larger *E. berryi* also inhabited coastal water areas (Figure 2). In autumn, most *E. berryi* remained in the Yushan fishing ground, adopting a wide distribution from Haizhou Bay to Wentai fishing grounds; both *E. berryi* biomass and individual sizes increased from the southern Yellow Sea to the East China Sea, with the longitudinal mean CPUE_w_ and AIW increasing in the order of 125°–126° E→124°–124.5° E→123.5° E→122°–123° E (Figure 2). During the winter, the highest *E. berryi* biomass occurred in the Wentai fishing ground, with a spatial distribution range that included fishing grounds from Haizhou Bay to Mindong; most *E. berryi* were found in a longitudinal range of 123°–123.5° E, with the mean AIW increasing in a longitudinal order of 125°–127° E→124°–124.5° E→123°–123.5° E→121.5° E (Figure 2). The *E. berryi* spawning season has been reported to occur from late April to July in Aichi, Japan [31], and from March to December in Taiwan [32], with Jolly et al. (2022) reporting that *E. berryi* adults were found from April to June in the southern and the Pacific Ocean side of mainland Japan [18]. In the current study, the highest CPUE_w_ values occurred in the order of autumn > winter > summer, with mean AIW decreasing from summer to autumn to winter (Table 1).

**Figure 3:**
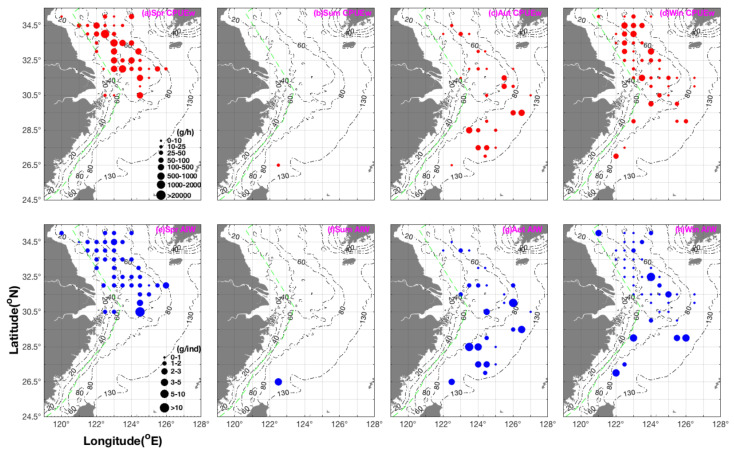
Seasonal distribution patterns of CPUE_w_ (red; grouped into 0–10, 10–25, 25–50, 50–100, 100–500, 500–1000, 1000–2000, and >20,000 g h^−1^) and AIW (blue; grouped into 0–1, 1–2, 2–3, 3–5, 5–10, and >10 g ind^−1^) for *Euprymna morsei*. Abbreviations: AIW, average individual weight; CPUE_w_, catch per unit effort by weight.

*E. morsei* was mainly distributed in the southern Yellow and northern East China Seas in spring, with smaller and larger individual sizes occurring in inshore and offshore areas, respectively. The latitudinal mean CPUE_w_ continuously increased in the order of 35° N→34.5° N→34° N→33.5° N, with the greatest biomass found at 122.5° E (Figure 3). In summer, this species was only recorded at one station (26.5° N 122.5° E). In autumn, smaller individuals were found in the latitudinal range of 34°–34.5° N, with smaller and larger individuals mainly found in northern and southern study areas, respectively; the greatest biomass occurred at 126°–127° E, with smaller individuals found at 125°–125.5° E (Figure 3). In winter, AIW ranged from 0.81 to 1.26 g ind^−1^ at 34.5° N and 1.2 to 4.844 g ind^−1^ at 27°–29° N, indicating a potential nursery function of these locations. The highest biomass occurred at 34° N and 122.5°–123.5° E (Figure 3). Our results indicated a relatively low biomass and numbers caught in autumn and winter, suggesting that *E. morsei* juveniles bury themselves under the substrate and, thus, avoid capture by trawl surveys. The order of the highest CPUE_w_ values was spring > winter > autumn, whereas that for mean AIW was summer > spring + autumn > winter (Table 1).

### 3.3. Model Evaluation and Suitable Habitat and Environmental Factors

The CTA, FDA, GAM, GBM, GLM, RF, XGBOOST, and ensemble models showed that variability in SSS was most important in determining the distribution of *E. berryi*, whereas the ANN algorithm suggested that variability in SBT was most important, and the MARS and SRE algorithms highlighted variability in SBS as being most important (Figure S1 in the Supplementary Material online). By contrast, all algorithms showed that variability in SST was the most important factor determining the distribution of *E. morsei* (Figure S1). Environmental factors, such as water temperature and other physico-chemical parameters, play a vital role in shaping species distribution and habitat suitability. Seasonal fluctuations in these parameters, as observed in previous studies (e.g., Naeem et al., 2011) [33], indicate that climate-induced changes could further impact habitat conditions for *E. berryi* and *E. morsei*. This highlights the need for continuous monitoring of environmental variables to better understand the ecological responses of these species under different climate scenarios. In terms of TSS and ROC, RF was the best model (Figures S2 and S3).

In terms of suitable habitat range, *E. berryi* expanded northward during autumn and winter compared with their location during the summer. By contrast, *E. morsei* was concentrated in the southern Yellow and northern East China Seas during the spring and winter compared with occurring throughout the study area during the summer and autumn (Figures S4–S6). The suitable habitat area for *E. morsei* extended to the south in winter to spring, becoming even more southerly during the summer and autumn (Figures S4–S6).

**Figure 4:**
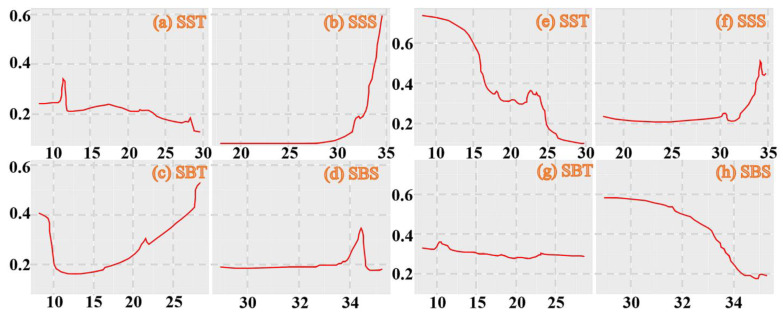
Responses of *Euprymna berryi* (**a**–**d**) and *Euprymna morsei* (**e**–**h**) to the environmental variables SST (10–30 °C), SSS (10–25 °C), SBT (20–35‰), and SBS (30–34‰). Abbreviations: SST, sea surface temperature; SBT, sea bottom temperature; SSS, sea surface salinity; SBS, sea bottom salinity.

The suitable environmental variables predicted for the study year for *E. berryi* were as follows: 10–30 °C SST, >28 °C SBT, >33‰ SSS, and ~34.5‰ SBS, whereas, for *E. morsei*, these were 10–15 °C SST, >10–25 °C SBT, >34‰ SSS, and 29–32‰ SBS (Figure 4).

**Figure 5:**
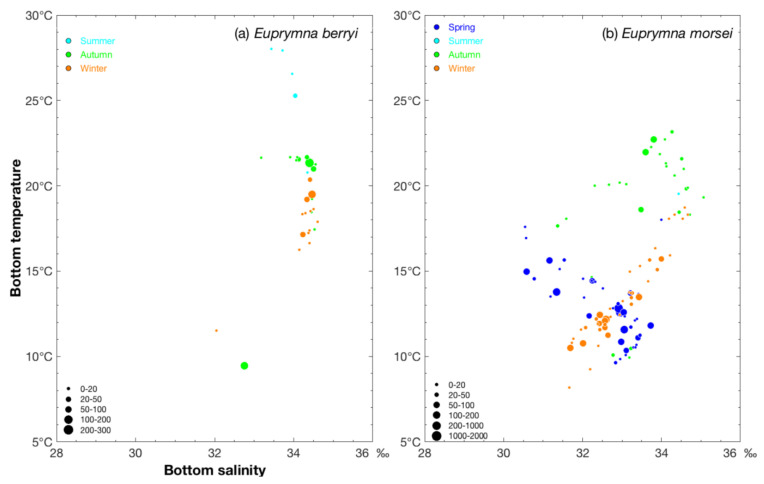
Relationship between SBS (‰) and SBT (°C) for CPUE_n_ sizes of *Euprymna berryi* (**a**) classified by group (0–20, 20–50, 50–100, 100–200, and 200–300 ind/h) and *Euprymna morsei* (**b**) classified by group (0–20, 20–50, 50–100, 100–200, 200–1000, and 1000–2000 ind/h). The data for spring, summer, autumn, and winter are denoted by blue, cyan, green, and brown–red solid circles, respectively. Abbreviations: CPUE_n_, catch per unit effort by number; SBS, sea bottom salinity; SBT, sea bottom temperature.

Choe (1966) [31] reported that the embryonic period of *E. berryi* was 20 days at 23.5–24.0 °C, with mature *E. berryi* observed spawning fertilized clutches repeatedly over a period of 100 days at 20 °C. In our study, *E. berryi* was most numerous in regions during the summer where the SBT was 25.29–28.02 °C and SBS was 33.43–34.04‰ compared with an SBT of 9.47 °C and SBS of 32.75‰ and SBT 20.99–21.69 °C and SBS of 34.07–34.50‰ during the autumn and an SBT of 17.13–20.36 °C and SBS of 34.23–34.46‰ during the winter (Figure 5a).

Watanabe (1997) [34] reported that the embryonic period of *E. morsei* was 32 days at 20 °C. In our study, *E. morsei* was most numerous in regions during the spring with an SBT of 12.59–12.83 °C and SBS of 32.95–32.91‰, during the summer in areas with a SBT of 19.54 °C and SBS of 34.43‰ compared with an SBT of 21.98–22.70 °C and SBS 33.80–33.60‰ in autumn and SBT of 10.51–13.49 °C and SBS 31.69–33.42‰ in winter (Figure 5b).

### 3.4. Habitat Predictions Under Different Climate Projections

Currently, the habitat area suitable for *E. morsei* is larger than that for *E. berryi*, with the latter mainly concentrated in the Mindong-Yushan fishing grounds. Under the SSP585-2050 scenario, the habitat area suitable for *E. berryi* would expand more northward. The habitat area suitable for *E. morsei* would retreat northward under the SSP370-2100 and SSP585-2100 scenarios (Table 3; Figures S7–S9).

Under the various climate scenarios, the area of habitat suitable for *E. morsei* would be smaller than that for *E. berryi*. Under SSP126-2050, SSP245-2050, and SSP585-2050, a reduction in area of <5% would occur for *E. berryi*, whereas a reduction of 5–10% would occur under scenarios SSP126-2100 and SSP370-2050, and a reduction of >10% would occur under scenarios SSP245-2100, SSP370-2100, and SSP585-2100 (Table 3).

By contrast, there would be more areas of suitable habitat for *E. morsei* than for *E. berryi* under scenarios SSP126-2050 and SSP245-2050 versus fewer areas of suitable habitat under scenarios SSP126-2100, SSP245-2100, SSP370-2050, SSP370-2100, SSP585-2050, and SSP585-2100. Increases in suitable habitat of <5% for *E. morsei* would occur under scenarios SSP126-2100, SSP245-2100, SSP370-2050, SSP370-2100, SSP585-2050, and SSP585-2100, whereas increases >5% would occur under SSP126-2050 and SSP245-2050. Increases in suitable habitat of <10% for *E. berryi* would occur under SSP126-2050, SSP126-2100, SSP245-2050, SSP370-2050, and SSP585-2050, whereas increases >10% would occur under SSP245-2100, SSP370-2100, and SSP585-2100 (Table 3).

In terms of total changes in area, increases in area of <10% for *E. berryi* would occur under SSP126-2050, SSP245-2050, and SSP585-2050, whereas reduced area percentages of <10% would occur under SSP126-2100, SSP245-2100, SSP370-2050, SSP370-2100, and SSP585-2100, with the percentage of reduced area being greatest under SSP585-2100. *E. morsei* would experience greater reductions in the amount of suitable habitat under all scenarios (>90% under SSP245-2100 and SSP585-2100; 50–90% under SSP245-2050, SSP370-2050, SSP370-2100, and SSP585-2100; and <50% under SSP126-2050 and SSP126-2100; Table 3).

In the East China Sea region, the distribution of *Sepia esculenta* was found to be more northerly than previously reported [35]. By the 2050s, the joint distribution areas of *Loliolus beka* and *Loliolus uyii* were predicted to expand to the central East China Sea and the southern Yellow Sea [36]. The habitat range of *Sepiella maindroni* will greatly decrease under the case of SSP585 [37]. The core habitat of *AmphiOctopus ovulum* is expected to expand to the northeast and southwest independently [38]. The annual mean habitat area of *AmphiOctopus fangsiao* will shrink significantly [39]. The habitat area range of *Abralia multihamata* will move poleward from spring to winter [40].

Both species are small in size and currently not commercially exploited. However, their small size would be economically advantageous because culture conditions would be required on a smaller scale with lower costs and fewer facility requirements compared with those used for pelagic and large-sized species. *E. berryi* and *E. morsei* are also suitable model species for reproductive studies because of their short lifespan (5–8 months). In addition, they are more easily maintained in laboratory conditions compared with other larger species. This study has some limitations. Specifically, the methods used in this study have potential risk of overfitting in predicting future distributions of the species under different climate scenarios [41]. This might cause bias about the spatial-temporal distribution patterns of both species owing to variability in trawling efficiency and others. Future work should focus on continuous population monitoring for both species, especially whether the population size of *E. berryi* alters compared with that of *E. morsei* in response to climate change, which can cause species succession and transformation of ecosystem functions in this study area.

## 4. Conclusions

There are several conclusions reached by our study. During the summer, larger *E. berryi* were found in shallower water areas, which then migrated northward during the autumn and winter. In autumn, there was a larger biomass of *E. berryi*, with increased numbers of larger individuals, in the southern Yellow Sea compared with East China Sea, concentrated in inshore areas to overwinter. During spring, *E. morsei* were concentrated for breeding in a small area, with an SBT of 12.59–12.83 °C and SBS of 32.95–32.91‰. During the autumn and winter, this species migrated southward from the southern Yellow and northern East China Seas. It might be that the fishing grounds of Haizhou Bay and Wentai-Yushan are nursery grounds for this species, whereas offshore areas provide overwintering grounds. SSS appeared to be the most important determinant of suitable habitat for *E. berryi*, whereas this was SST for *E. morsei*. In this study, we used ensemble models produced by very often used algorithms to identify the seasonal spatial distribution patterns across the seasons and distribution range variations in both species under different climate scenarios. In the future, we will discuss the relationship between single algorithms and an ensemble model in the case of the available verified survey data. The biological ecological information of both species obtained in this study can offer insights into how small-size squids are varying corresponding to climate changes and, thus, offer key insights to fishery managers about ecosystem-based management incorporating into climate changes. Finally, we appeal that ocean governance should consider climate change and forecasting to drive transformative, sustainable, and inclusive ocean care.

## Figures and Tables

**Table 1:** Seasonal total biomass density (CPUE_w_) and density (CPUE_n_) of *Euprymna berryi* and *Euprymna morsei* in the study area.

Season	*Euprymna berryi*		*Euprymna morsei*	
	CPUE_w_ (g·h^−1^)	CPUE_n_ (ind·h^−1^)	CPUE_w_ (g·h^−1^)	CPUE_n_ (ind·h^−1^)
Spring	-	-	8555.88	5243
Summer	483.12	48	11.1	3
Autumn	3328.73	636	986.4	662
Winter	1612.86	431	1664.7	1732

**Table 2:** Seasonal variation in environmental factors, biological indicators, and suitable habitat distribution range for *Euprymna berryi* and *Euprymna morsei* in the study area from autumn 2018 to summer 2019 ^a^.

Factor	*Euprymna berryi*			*Euprymna morsei*			
	Summer	Autumn	Winter	Spring	Summer	Autumn	Winter
Depth (m)	55–84	58–107	38–126	13–82	104	16–112	16–114
SST (°C)	26.39–28.56	18.92–23.66	11.51–20.42	12.85–17.35	26.11	18–24.56	8.21–20.52
SBT (°C)	20.78–28.02	9.47–21.69	11.52–20.36	9.64–17.99	19.54	9.92–23.15	8.17–18.73
SSS (‰)	31.92–34.02	31.7–34.23	32.06–34.52	30.21–33.49	34.07	30.49–34.29	31.5–34.51
SBS (‰)	33.43–34.34	32.75–34.55	32.04–34.61	30.55–33.99	34.43	31.37–35.07	31.67–34.66
SSDO (mg/L)	5.22–6.44	/	7.35–8	7.93–8.63	6.06	/	7.33–9.08
SBDO (mg/L)	4.25–6.62	/	7.36–7.97	7.71–9.23	5.3	/	7.58–9.06
Mean CPUE_w_ at collection stations (g/h)	80.52	256.06	94.87	213.9	11.1	36.53	38.71
CPUE_w_ range (g/h)	25.2–154.96	1.35–2181.6	3.2–494.6	1.34–3203.2	11.1	2.4–178.82	0.5–297.6
Mean CPUE_n_ at collection stations (ind/h)	8	48.92	25.35	131.08	3	24.52	40.28
CPUE_n_ range (ind/h)	3–21	1–288	1–161	1–1824	3	1–124	1–272
Mean AIW (g/ind)	12.77	7.33	4.35	1.7	3.7	1.74	1.33
AIW range (g/ind)	6.3–26.33	0.75–20.9	0.8–13	0.71–11.29	3.7	0.53–5.56	0.16–8.47
Suitable habitat range	26.55°–29.35° N, 121.55°–126.95° E	26.55°–32.65° N, 121.55°–126.95° E	26.55°–32.25° N, 120.05°–126.95° E	28.45°–34.95° N, 120.05°–126.45° E	26.55°–34.95° N, 120.05°–125.65° E	26.55°–34.95° N, 120.05°–126.95° E	29.65°–34.95° N, 120.05°–126.25° E

^a^ Abbreviations: SST, sea surface temperature; SBT, sea bottom temperature; SSS, sea surface salinity; SBS, sea bottom salinity; SSDO, sea surface dissolved oxygen; SBDO, sea bottom dissolved oxygen; CPUE_w_, catch per unit effort by weight; CPUE_n_, catch per unit effort by number; AIW, average individual weight.

**Table 3:** Percentages of habitat loss, gain, and overall habitat (gain minus loss) and most suitable habitat range for *Euprymna berryi* and *Euprymna morsei* under different climate scenarios.

Climate Scenario		Loss	Gain	Total	Suitable Habitat Range
Current	*E. berryi*	/	/	/	26.55° N–29.35° N, 121.45° E–126.95° E
	*E. morsei*	/	/	/	26.55° N–34.95° N, 120.05° E–126.95° E
SSP126-2050	*E. berryi*	−0.681%	5.917%	5.236%	26.55° N–29.55° N, 121.55° E–126.95° E
	*E. morsei*	−61.571%	18.09%	−43.481%	26.55° N–34.95° N, 120.05° E–126.95° E
SSP126-2100	*E. berryi*	−9.751%	7.649%	−2.102%	26.55° N–29.35° N, 121.55° E–126.95° E
	*E. morsei*	−51.547%	4.734%	−46.813%	26.55° N–34.95° N, 120.05° E–126.95° E
SSP245-2050	*E. berryi*	−0.584%	4.885%	4.301%	26.55° N–29.55° N, 121.45° E–126.95° E
	*E. morsei*	−61.412%	11.267%	−50.145%	26.55° N–34.95° N, 120.05° E–126.95° E
SSP245-2100	*E. berryi*	−19.015%	10.568%	−8.447%	26.55° N–29.35° N, 121.55° E–126.95° E
	*E. morsei*	−91.775%	0.344%	−91.431%	27.05° N–34.95° N, 120.05° E–126.25° E
SSP370-2050	*E. berryi*	−5.605%	3.503%	−2.102%	26.55° N–29.45° N, 121.45° E–126.95° E
	*E. morsei*	−56.043%	0.45%	−55.594%	26.55° N–34.95° N, 120.05° E–126.95° E
SSP370-2100	*E. berryi*	−24.912%	18.276%	−6.637%	26.55° N–29.35° N, 121.55° E–126.95° E
	*E. morsei*	−70.114%	0%	−70.114%	29.55° N–34.95° N, 120.05° E–126.25° E
SSP585-2050	*E. berryi*	−1.012%	4.301%	3.289%	26.55° N–30.15° N, 121.55° E–126.95° E
	*E. morsei*	−68.659%	3.227%	−65.432%	26.55° N–34.95° N, 120.05° E–126.95° E
SSP585-2100	*E. berryi*	−35.5%	18.606%	−16.894%	26.55° N–29.45° N, 121.75° E–126.95° E
	*E. morsei*	−90.743%	0%	−90.743%	29.65° N–34.95° N, 120.05° E–126.25° E

## Data Availability

The data sets used and/or analyzed during the current study are available from the corresponding author on reasonable request.

## Supplementary Materials

The following supporting information can be downloaded at: https://www.mdpi.com/article/10.3390/biology14040327/s1, Figure S1. Box plot of variable important against environmental variables including sea bottom salinity (SBS), sea bottom temperature (SBT), sea surface salinity (SSS), and sea surface temperature (SST), of *Euprymna berryi* (right panels: a–k) and *Euprymna morsei* (left panels: l–v), produced by the algorithms of (a,l) artificial neural network (ANN), (b,m) classification tree analysis (CTA), (c,n) flexible discriminant analysis (FDA), (d,o) generalized additive model (GAM), (e,p) generalized boosting model (GBM), (f,q) generalized linear model (GLM), (g,r) multiple adaptive regression splines (MARS), (h,s) random forest (RF), (i,t) surface range envelope (SRE), (j,u) extreme gradient boosting training (XGBOOST), and (k,v) ensemble model. Figure S2. Calibration percentage (%) of TSS and ROC for the species *Euprymna berryi* (upper panels) and *Euprymna morsei* (bottom panels) in different algorithms of artificial neural network (ANN), classification tree analysis (CTA), flexible discriminant analysis (FDA), generalized additive model (GAM), generalized boosting model (GBM), generalized linear model (GLM), multiple adaptive regression splines (MARS), random forest (RF), surface range envelope (SRE), and extreme gradient boosting training (XGBOOST). Figure S3. The ratio values of TSS against ROC for the species *Euprymna berryi* (left panel) and *Euprymna morsei* (right panel) with x-direction and y-direction error bars produced by different methods, including artificial neural network (ANN), classification tree analysis (CTA), flexible discriminant analysis (FDA), generalized additive model (GAM), generalized boosting model (GBM), generalized linear model (GLM), multiple adaptive regression splines (MARS), random forest (RF), surface range envelope (SRE), and extreme gradient boosting training (XGBOOST), displayed by the colors of black, blue, green, cyan, red, pink, dark yellow, dark blue, purple, and red–brown. Figure S4. Seasonal spatial distribution patterns of *Euprymna berryi* (upper panels: a–d) and *Euprymna morsei* (bottom panels: e–h) in spring to winter in the study area predicted with the ensemble model consisting of the algorithms of artificial neural network (ANN), classification tree analysis (CTA), flexible discriminant analysis (FDA), generalized additive model (GAM), generalized boosting model (GBM), generalized linear model (GLM), multiple adaptive regression splines (MARS), random forest (RF), surface range envelope (SRE), and extreme gradient boosting training (XGBOOST). The color of blue to green indicates the range from low to high suitability independently. Figure S5. The predicted habitat suitability for *Euprymna berryi* in different seasons (spring, summer, autumn, winter). The red and blue area indicates the suitability index of 0.7–1 and 0–0.7 independently. Figure S6. The predicted habitat suitability for *Euprymna morsei* in different seasons (spring, summer, autumn, winter). The red and blue area indicates the suitability index of 0.7–1 and 0–0.7 independently. Figure S7. Predicted spatial habitat distribution patterns of *Euprymna berryi* (a–i) and *Euprymna morsei* (j–r) in the cases of annual mean habitat, SSP1-2.6 in 2050, SSP1-2.6 in 2100, SSP2-4.5 in 2050, SSP2-4.5 in 2100, SSP3-7.0 in 2050, SSP3-7.0 in 2100, SSP5-8.5 in 2050, and SSP5-8.5 in 2100. The bar colored in blue to green indicates the range from low to high suitability. Figure S8. The predicted habitat suitability for *Euprymna berryi* in different climate scenarios: (a) current; (b) SSP1-2.6 in 2050; (c) SSP1-2.6 in 2100; (d) SSP2-4.5 in 2050; (e) SSP2-4.5 in 2100; (f) SSP3-7.0 in 2050; (g) SSP3-7.0 in 2100; (h) SSP5-8.5 in 2050; and (i) SSP5-8.5 in 2100. The red and blue area indicates the suitability index of 0.7–1 and 0–0.7 independently. Figure S9. The predicted habitat suitability for *Euprymna morsei* in different climate scenarios: (a) current; (b) SSP1-2.6 in 2050; (c) SSP1-2.6 in 2100; (d) SSP2-4.5 in 2050; (e) SSP2-4.5 in 2100; (f) SSP3-7.0 in 2050; (g) SSP3-7.0 in 2100; (h) SSP5-8.5 in 2050; and (i) SSP5-8.5 in 2100. The red and blue area indicates the suitability index of 0.7–1 and 0–0.7 independently.
