# Supplementary material for: Changes in Seasonal Spatial Distribution Patterns of Euprymna berryi and Euprymna morsei: The Current and Predictions Under Climate Change Scenarios
Source: Biology (Basel). 2025 Mar 24;14(4):327. doi: 10.3390/biology14040327 (PMC12024566; doi:10.3390/biology14040327)
Supplement: Supplementary file 1 [file biology-14-00327-s001.zip › biology-3513427-supplementary.pdf]

Supplementary File

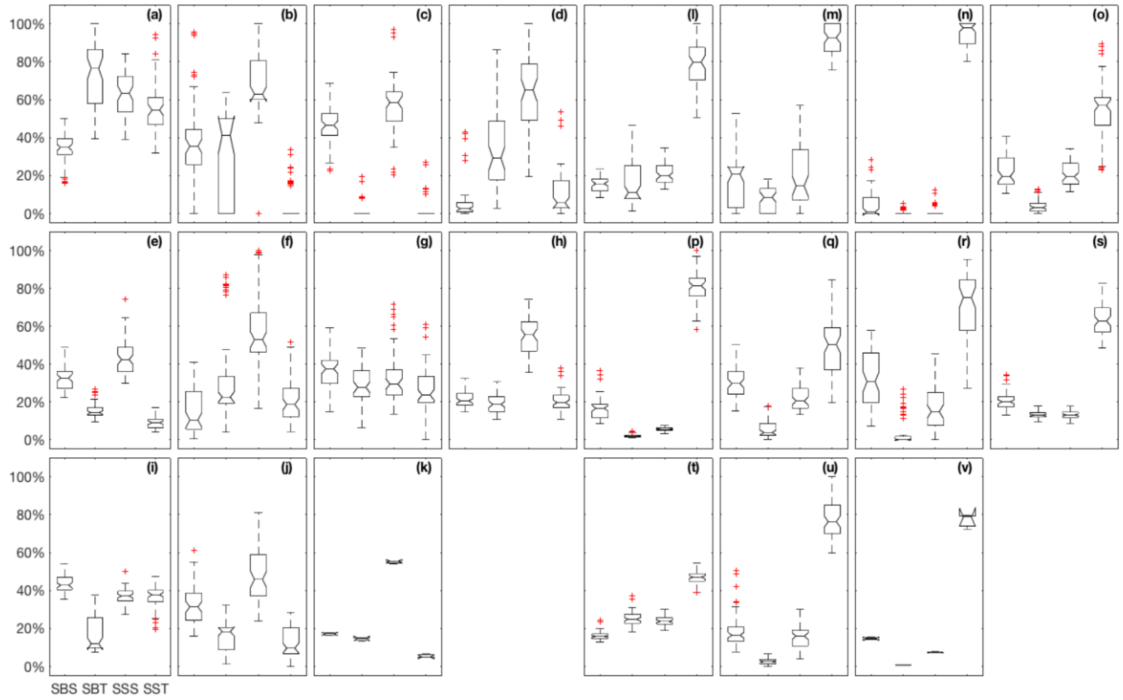

Figure S1. Box plot of variable important against environmental variables including sea bottom salinity (SBS), sea bottom temperature (SBT), sea surface salinity (SSS), and sea surface temperature (SST), of *Euprymna berryi* (right panels: a–k) and *Euprymna morsei* (left panels: l–v), produced by the algorithms of (a,l) artificial neural network (ANN), (b,m) classification tree analysis (CTA), (c,n) flexible discriminant analysis (FDA), (d,o) generalized additive model (GAM), (e,p) generalized boosting model (GBM), (f,q) generalized linear model (GLM), (g,r) multiple adaptive regression splines (MARS), (h,s) random forest (RF), (i,t) surface range envelope (SRE), (j,u) extreme gradient boosting training (XGBOOST), (k,v) ensemble model.

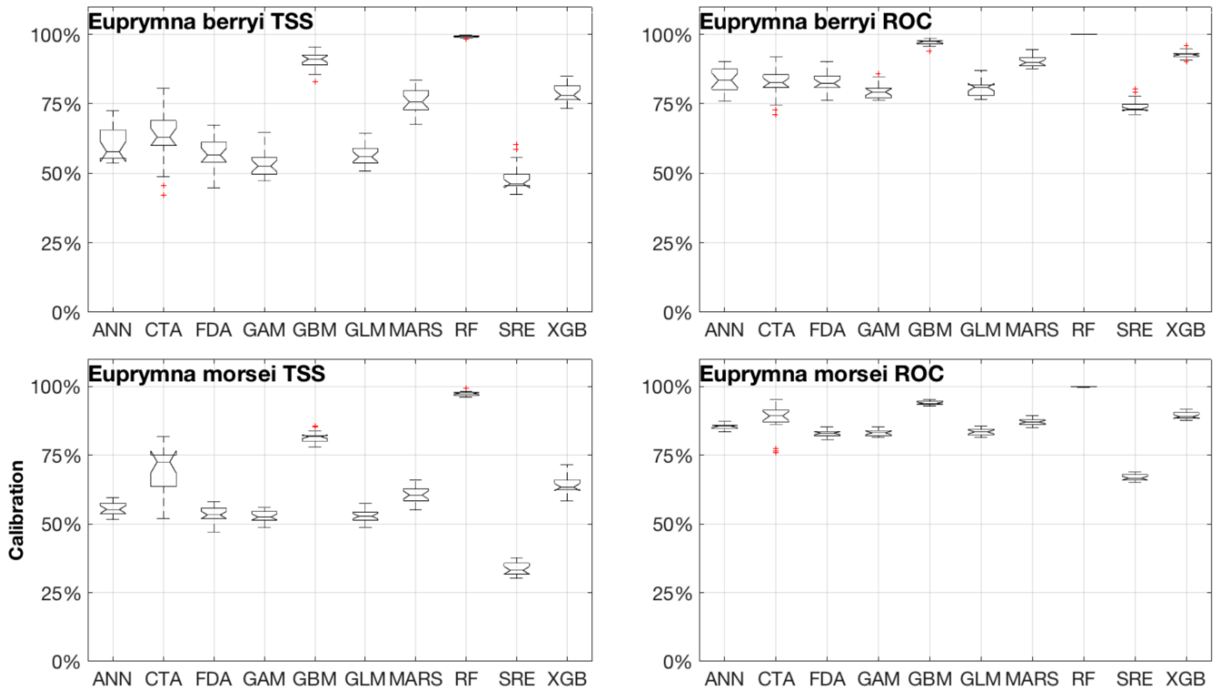

Figure S2. Calibration percentage (%) of TSS and ROC for the species *Euprymna berryi* (upper panels) and *Euprymna morsei* (bottom panels) in different algorithms of artificial neural network (ANN), classification tree analysis (CTA), flexible discriminant analysis (FDA), generalized additive model (GAM), generalized boosting model (GBM), generalized linear model (GLM), multiple adaptive regression splines (MARS), random forest (RF), surface range envelope (SRE), extreme gradient boosting training (XGBOOST).

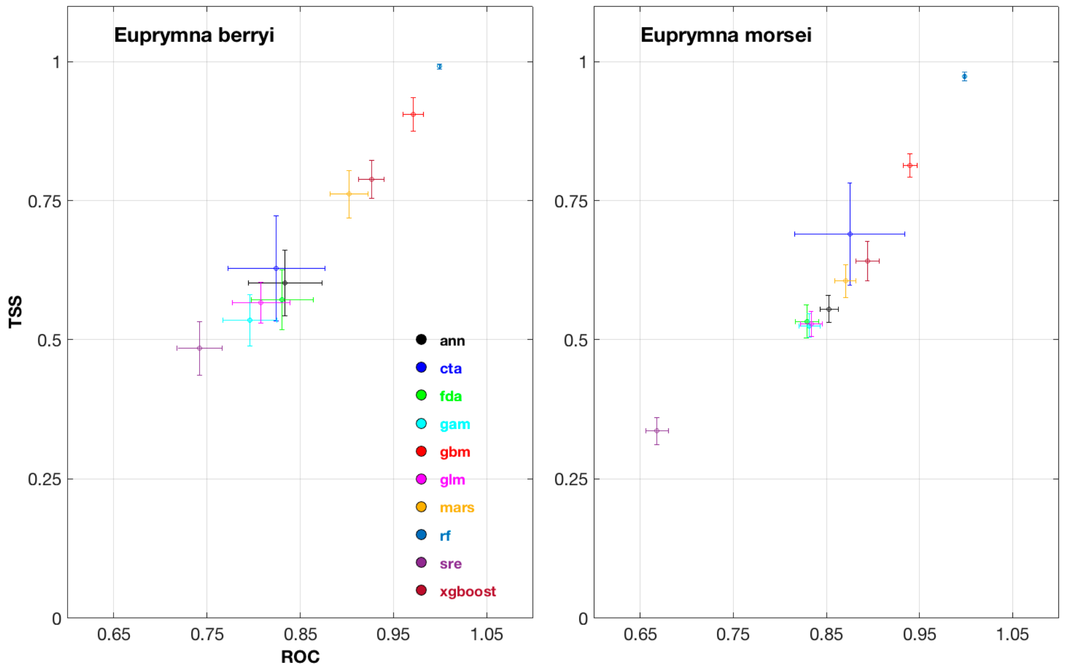

Figure S3. The ratio values of TSS against ROC for the species *Euprymna berryi* (left panel) and *Euprymna morsei* (right panel) with x-direction and y-direction error bars produced

by different methods including artificial neural network (ANN), classification tree analysis (CTA), flexible discriminant analysis (FDA), generalized additive model (GAM), generalized boosting model (GBM), generalized linear model (GLM), multiple adaptive regression splines (MARS), random forest (RF), surface range envelope (SRE), extreme gradient boosting training (XGBOOST), displayed by the colors of black, blue, green, cyan, red, pink, dark yellow, dark blue, purple, and red-brown.

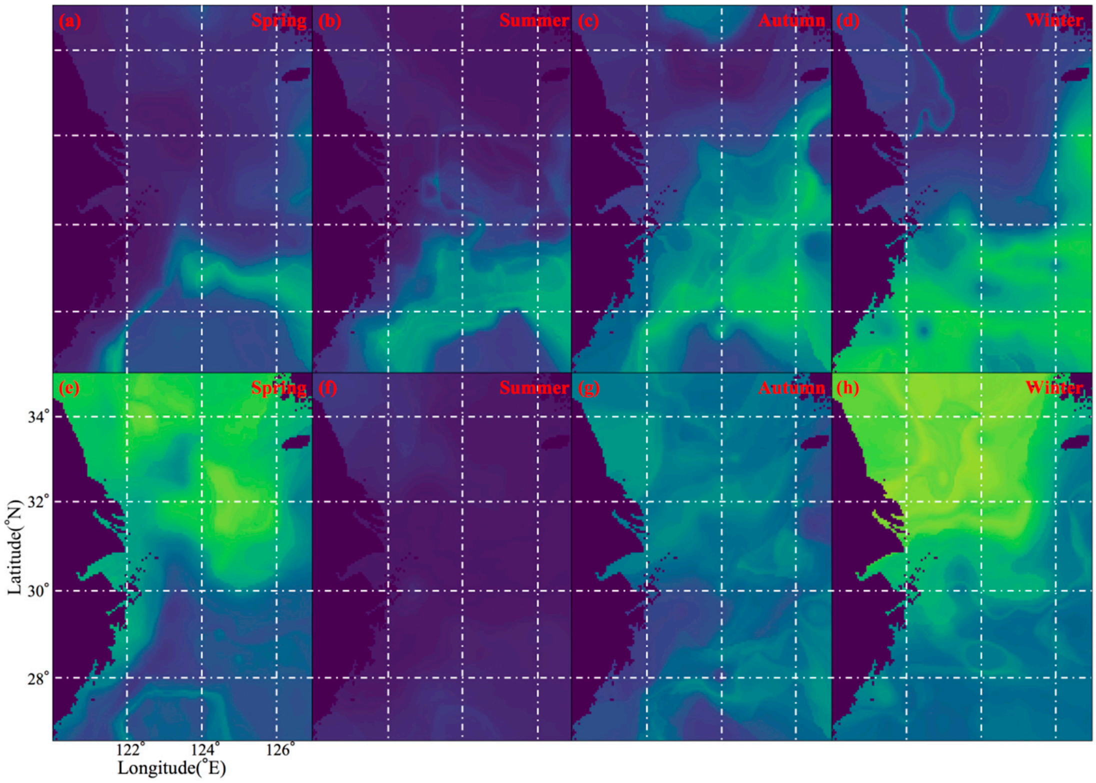

Figure S4. Seasonal spatial distribution patterns of *Euprymna berryi* (upper panels: a–d) and *Euprymna morsei* (bottom panels: e–h) in spring to winter in the study area predicted with the ensemble model consisting of the algorithms of artificial neural network (ANN), classification tree analysis (CTA), flexible discriminant analysis (FDA), generalized additive model (GAM), generalized boosting model (GBM), generalized linear model (GLM), multiple adaptive regression splines (MARS), random forest (RF), surface range envelope (SRE), and extreme gradient boosting training (XGBOOST). The color of blue to green indicates the range from low to high suitability independently.

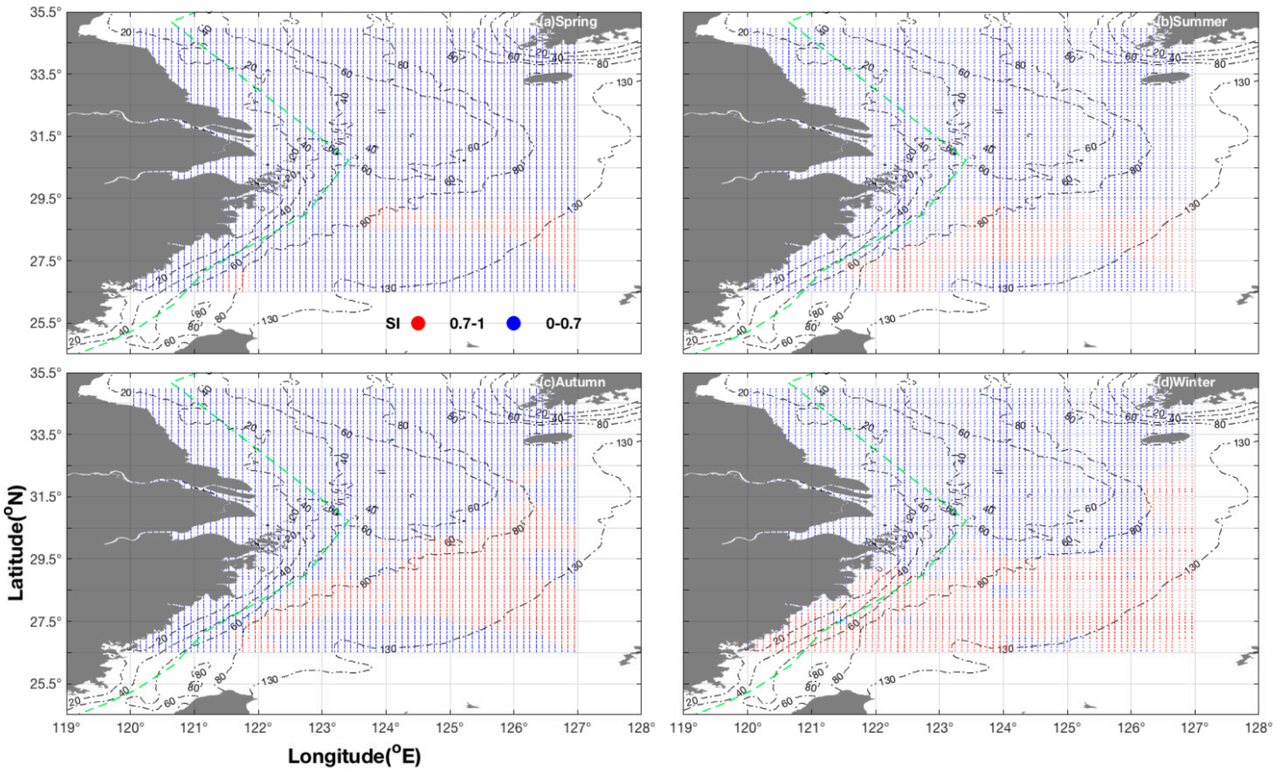

Figure S5. The predicted habitat suitability for *Euprymna berryi* in different seasons (spring, summer, autumn, winter). The red and blue area indicate the suitability index of 0.7-1 and 0-0.7 independently.

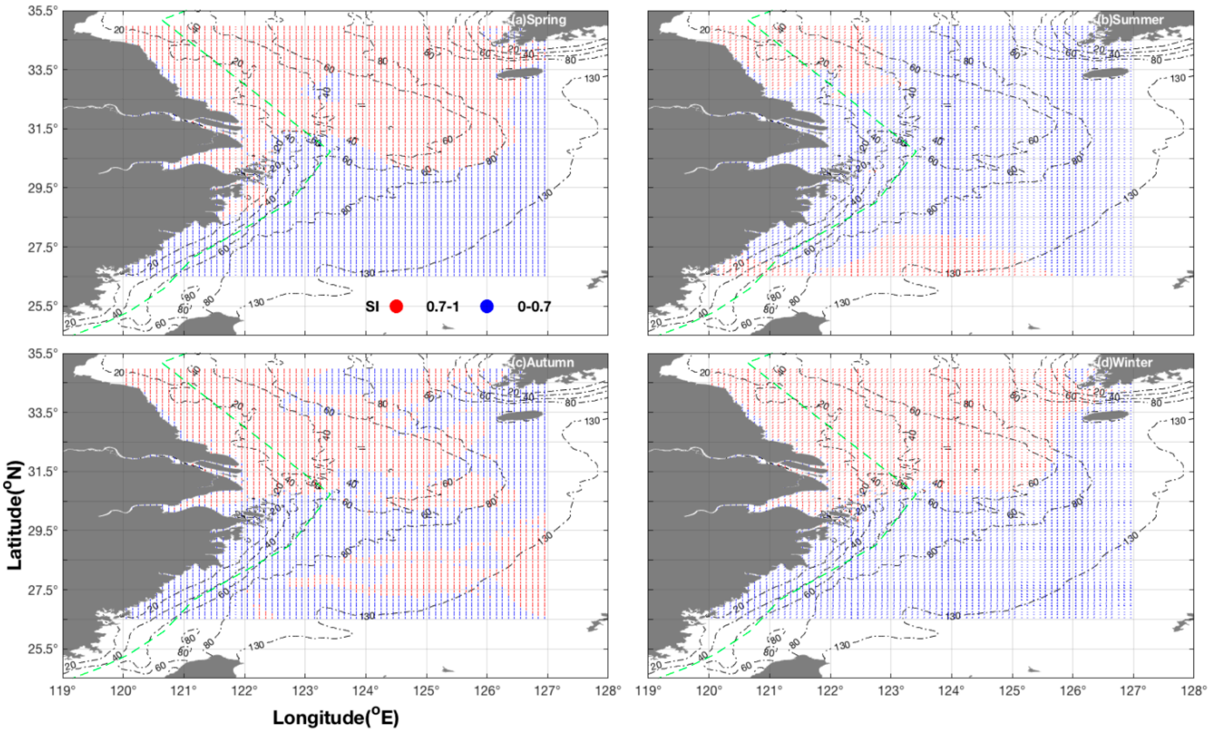

Figure S6. The predicted habitat suitability for *Euprymna morsei* in different seasons (spring, summer, autumn, winter). The red and blue area indicate the suitability index of 0.7-1 and 0-0.7 independently.

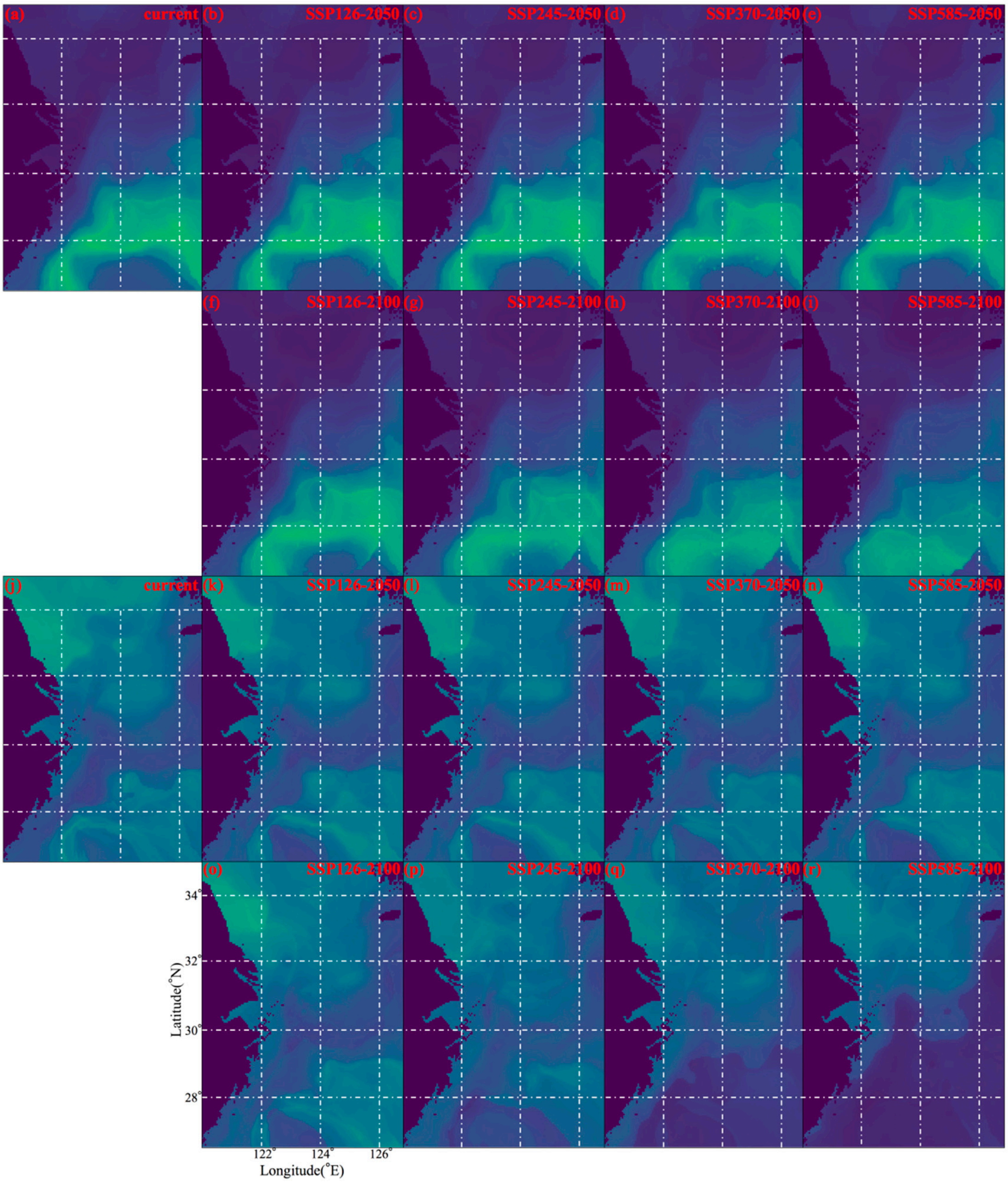

Figure S7. Predicted spatial habitat distribution patterns of *Euprymna berryi* (a–i) and *Euprymna morsei* (j–r) in the cases of annual mean habitat, SSP1–2.6 in 2050, SSP1–2.6 in 2100, SSP2–4.5 in 2050, SSP2–4.5 in 2100, SSP3–7.0 in 2050, SSP3–7.0 in 2100, SSP5–8.5 in 2050, SSP5–8.5 in 2100. The bar colored in blue to green indicates the range from low to high suitability.

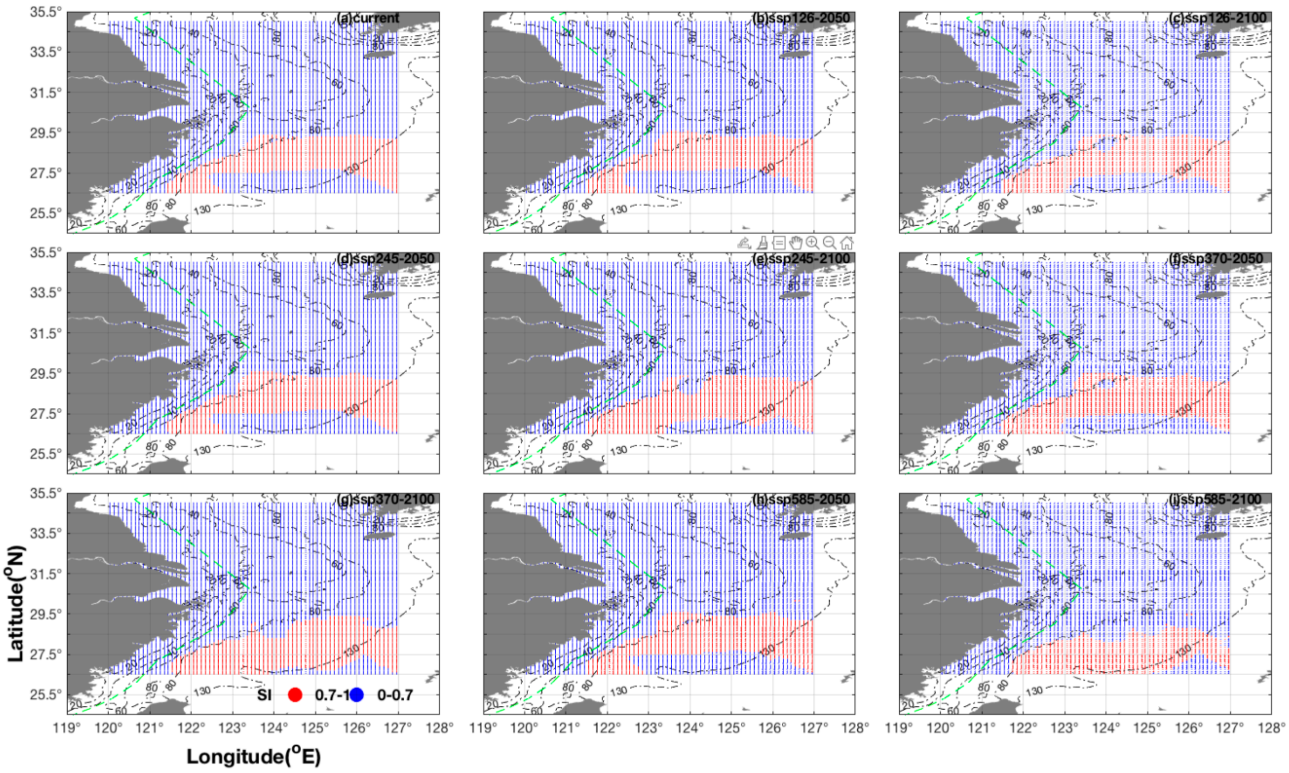

Figure S8. The predicted habitat suitability for *Euprymna berryi* in different climate scenarios (a) current; (b) SSP1-2.6 in 2050; (c) SSP1-2.6 in 2100; (d) SSP2-4.5 in 2050; (e) SSP2-4.5 in 2100; (f) SSP3-7.0 in 2050; (g) SSP3-7.0 in 2100; (h) SSP5-8.5 in 2050; and (i) SSP5-8.5 in 2100. The red and blue area indicates the suitability index of 0.7-1 and 0-0.7 independently.

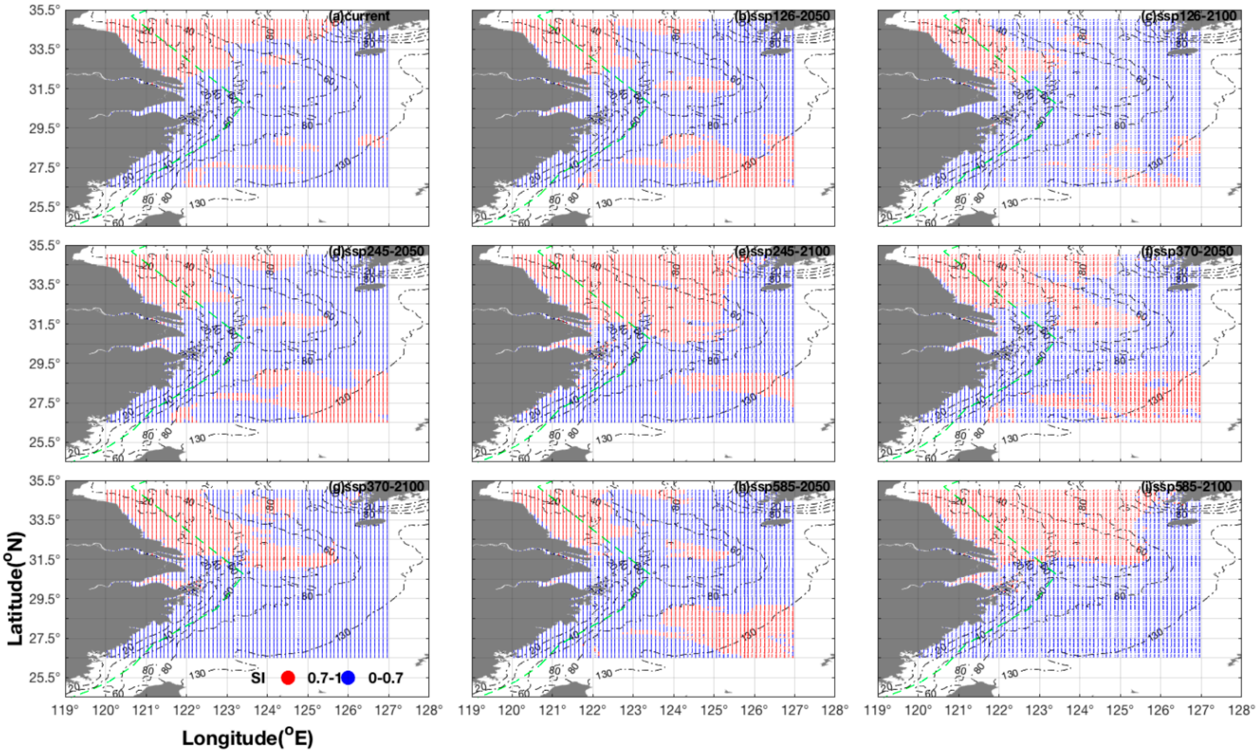

Figure S9. The predicted habitat suitability for *Euprymna morsei* in different climate scenarios (a) current; (b) SSP1-2.6 in 2050; (c) SSP1-2.6 in 2100; (d) SSP2-4.5 in 2050; (e) SSP2-4.5 in 2100; (f) SSP3-7.0 in 2050; (g)

SSP3-7.0 in 2100; (h) SSP5-8.5 in 2050; and (i) SSP5-8.5 in 2100. The red and blue area indicates the suitability index of 0.7-1 and 0-0.7 independently.
